# Overexpression of Rice Os*S1Fa1* Gene Confers Drought Tolerance in Arabidopsis

**DOI:** 10.3390/plants10102181

**Published:** 2021-10-14

**Authors:** Sung-Il Kim, Kyu Ho Lee, Jun Soo Kwak, Dae Hwan Kwon, Jong Tae Song, Hak Soo Seo

**Affiliations:** 1 Department of Agriculture, Forestry and Bioresources, Research Institute of Agriculture and Life Sciences, Seoul National University, Seoul 08826, Korea; icuts@snu.ac.kr (S.-I.K.); leehotl@snu.ac.kr (K.H.L.); kchhy88@snu.ac.kr (J.S.K.); kdh9495@snu.ac.kr (D.H.K.); 2 Department of Applied Biosciences, Kyungpook National University, Daegu 41566, Korea; jtsong68@knu.ac.kr

**Keywords:** small protein, rice, OsS1Fa1, drought stress, post-translational modification

## Abstract

Small peptides and proteins play critical regulatory roles in plant development and environmental stress responses; however, only a few of these molecules have been identified and characterized to date because of their poor annotation and other experimental challenges. Here, we present that rice (*Oryza sativa* L.) OsS1Fa1, a small 76-amino acid protein, confers drought stress tolerance in *Arabidopsis thaliana*. *OsS1Fa1* was highly expressed in leaf, culm, and root tissues of rice seedlings during vegetative growth and was significantly induced under drought stress. *OsS1Fa1* overexpression in Arabidopsis induced the expression of selected drought-responsive genes and enhanced the survival rate of transgenic lines under drought. The proteasome inhibitor MG132 protected the OsS1Fa1 protein from degradation. Together, our data indicate that the small protein OsS1Fa1 is induced by drought and is post-translationally regulated, and the ectopic expression of *OsS1Fa1* protects plants from drought stress.

## 1. Introduction

Abiotic stresses such as drought, salinity, cold, and heat induce biochemical and molecular changes that affect plant growth and crop yields [1,2]. To survive under these stress conditions, plants activate various cell signaling pathways such as the production of antioxidants, the induction of stress-related proteins, and the accumulation of compatible solutes [1,3].

Drought refers to low water availability for an extended period of time. At the molecular level, plant drought resistance is a complex process involving many genes and signaling pathways that modify several physiological, morphological, and molecular responses. The perception of external drought stress stimuli by sensors located at the plant cell membrane induces the expression of a large number of genes with diverse functions, resulting in drought adaptation [4,5].

Drought-responsive proteins can be categorized into three groups: transcription and signaling cascade-related proteins such as transcription factors and protein kinases; protein- and membrane-protecting proteins such as the late embryogenesis abundant (LEA) protein and antioxidants; and water and ion uptake and transport-related proteins such as aquaporins and sugar transporters [4,6,7,8]. Thousands of drought-responsive genes have been identified using next-generation sequencing (NGS) approaches such as RNA sequencing (RNA-seq), and the functions of many of these genes have been characterized to date [9,10].

S1Fa is a spinach (*Spinacia oleracea* L.) nuclear protein that binds to the *cis*-element of the Site 1 binding site, one of three binding sites (Site 1, 2, and 3) located within the promoter of the nuclear gene *rps1*, which encodes the plastid ribosomal protein cS [11]. S1Fa is a small protein, with only 76 amino acids (aa), and is different from the Site 1 Factor (S1F) protein, which has been characterized in spinach leaf extracts as a 28–30 kDa protein [12]. The predicted amino acid sequence of S1Fa contains a nuclear localization signal (NLS) peptide and a DNA recognition motif [13], supporting its function as a transcription factor. The spinach *S1Fa* gene is expressed at higher levels in roots and etiolated seedlings than in green leaves, indicating that S1Fa binds to the *rps1* promoter to repress its expression specifically in roots and etiolated plants [13].

S1Fa is highly conserved among dicots and monocots. Although there are no more than five S1Fa-like protein families in several plant species, including rice (*Oryza sativa* L.), soybean (*Glycine max* L.), tomato (*Solanum lycopersicum* L.), and Arabidopsis, 126 S1Fa-like protein families have been identified in *Arachis duranensis*, and genes encoding the S1Fa proteins belonging to these 126 families are more highly expressed in roots and etiolated seedlings than in green leaves, similar to spinach *S1Fa* [14]. These findings suggest that S1Fa plays important roles in plant growth and development, including in responses to environmental stress. Nevertheless, the biochemical, molecular, and physiological functions of S1Fa have not yet been elucidated.

According to previous studies, small proteins and peptides are involved in plant growth, development, reproduction, and environmental stress responses [15,16,17,18]. Nevertheless, compared with larger proteins, the roles of small proteins in plant processes remain poorly understood. In this study, we provide evidence showing that the rice small protein OsS1Fa1, a homolog of spinach S1Fa, participates in plant survival under drought, and its stability is regulated by the ubiquitination pathway.

## 2. Materials and Methods

### 2.1. Plant Growth Conditions and Stress Treatments

Rice (*Oryza sativa* L. cv. Nipponbare) seeds were germinated in soil in a growth chamber at 28 °C under a 14 h light/10 h dark cycle, and seedlings were grown under the same conditions for 3 weeks. In the drought stress treatment, watering of seedlings was withheld for 10 d, whereas in the mock treatment, seedlings were watered every day for 10 d. After 10 d, samples were harvested, frozen in liquid nitrogen, and stored at −70 °C until needed for further analysis. Plants of *Arabidopsis thaliana* ecotype Columbia (Col-0; wild type [WT]) and *OsS1Fa1* overexpression lines were grown in a growth chamber at 22 °C under a 16 h light/8 h dark cycle on Murashige and Skoog (MS) medium supplemented with 0.5 g/L MES, 10 g/L sucrose, and 0.75% agar. To test the drought stress tolerance of WT and transgenic Arabidopsis plants, seeds of these genotypes were cold-stratified on wet soil at 4 °C in the dark for 3 d, and seedlings were grown at 22 °C under a 16 h light/8 h dark photoperiod, without watering, for 28 d. After the drought stress treatment, plants were rewatered for 5 d and then photographed.

### 2.2. Production of Recombinant Proteins

To produce His_6_-OsS1Fa1, full-length *OsS1Fa1* cDNA was cloned into the pET28a vector (Novagen, Madison, WI, USA). The resultant construct was transformed into *Escherichia coli* BL21/DE3 (pLysS) cells. Then, isopropyl-β-d-thiogalactoside (IPTG) was added to the transformed *E. coli* cell culture to induce the expression of the fusion protein. To extract the His_6_-OsS1Fa1 recombinant protein, bacteria were lysed in a buffer containing 50 mM NaH_2_PO_4_ (pH 8.0), 300 mM NaCl, 1% Triton X-100, 1 mM imidazole, 5 mM DTT, 2 mM PMSF, and a proteinase inhibitor cocktail (Roche, Basel, Switzerland). The extracted recombinant protein was then purified using Ni^2+^-nitrilotriacetate (Ni^2+^-NTA) resin (Qiagen, Hilden, Germany), according to the manufacturer’s instructions. Primers used for plasmid construction are listed in Table S1.

### 2.3. RT-qPCR Analysis

To evaluate the effect of drought stress on *OsS1Fa1* expression, the mock- and drought-treated rice leaf samples were ground thoroughly to obtain a fine powder. Total RNA was isolated from the ground tissue using the FavorPrep^TM^ Plant Total RNA Mini Kit (Favorgen, Ping-Tung, Taiwan) and then reverse transcribed to produce cDNA using ReverTra Ace^®^ qPCR RT Master Mix, with gDNA Remover (TOYOBO, Osaka, Japan). Then, the cDNA template was amplified by RT-qPCR on LightCycler^®^480 using the KAPA SYBR^®^ FAST qPCR Master Mix (2X) Kit (Kapa Biosystems, Wilmington, NC, USA) and *OsS1Fa1*-specific primers. The *Ubiquitin 5* (*UBQ5*) gene was used as an internal reference.

To examine the level of *OsS1Fa1* expression in rice seedlings during vegetative growth, total RNA was isolated from the shoot, leaf, culm, and root tissues of rice seedlings harvested at five different vegetative growth stages and amplified by RT-qPCR.

To examine the transcript levels of drought-responsive genes in Arabidopsis, total RNA was isolated from the leaves of 14-d-old WT and *OsS1Fa1*-overexpressing plants, and RT-qPCR was carried out using gene-specific primers. Primers for *Actin* (internal control) were added to the RT-qPCR reaction together with other gene-specific primers.

All experiments were repeated three times, with three replicates per sample. Primers used for all RT-qPCR assays are listed in Table S2.

### 2.4. Antibody Production and Western Blotting

The recombinant His_6_-OsS1Fa1 protein was purified using the Ni^2+^-NTA affinity column, according to the manufacturer’s instructions (Qiagen), and the concentration of the purified protein was measured with the Bradford assay [19]. The anti-OsS1Fa1 antibody was produced by subcutaneous injection of the recombinant protein into rabbits. To examine the level of OsS1Fa1 in rice and Arabidopsis, total proteins were extracted from the required samples and separated by sodium dodecyl sulfate-polyacrylamide gel electrophoresis (SDS-PAGE) on 12% acrylamide gel. The OsS1Fa1 protein was then detected by Western blotting with anti-OsS1Fa1 antibody.

### 2.5. Production and Characterization of Transgenic Arabidopsis Lines

To generate transgenic Arabidopsis lines overexpressing *OsS1Fa1*, the full-length cDNA of *OsS1Fa1* was cloned into the pBA002 vector under the control of the cauliflower mosaic virus (CaMV) *35S* promoter. The resulting *35S-OsS1Fa1* construct was introduced into *Agrobacterium tumefaciens* strain LBA4404, which was further used to transform Arabidopsis via the floral dipping method [20]. The expression level of *OsS1Fa1* was examined in transgenic plants by RT-qPCR (as described above), and the protein level of OsS1Fa1 was examined in the leaves of 14-d-old WT and transgenic plants by Western blotting with anti-OsS1Fa1 antibody.

### 2.6. Prediction of Conserved Motifs in the OsS1Fa1 Protein

Conserved motifs in the OsS1Fa1 protein were predicted based on its amino acid sequences using the QUARK computer algorithm [21] (https://zhanglab.Ccmb.med.umich.edu/QUARK/, accessed on 2 October 2018).

### 2.7. Effect of MG132 on OsS1Fa1 Protein Level and Stability

To perform a cell-free degradation assay, leaves of 10-d-old soil-grown rice seedlings were ground in liquid nitrogen and resuspended in a buffer containing 25 mM Tris-HCl (pH 7.5), 10 mM NaCl, 10 mM MgCl_2_, 4 mM PMSF, 5 mM DTT, and 10 mM ATP. Cell debris was pelleted by centrifugation, and equal volumes of the supernatant were aliquoted into individual tubes. Then, purified His_6_-OsS1Fa1 and 50 µM MG132 (Calbiochem, San Diego, CA, USA) were added to the protein extract in each tube, and samples were incubated at room temperature for 4 h. The reaction was stopped by adding an equal volume of 2× SDS sample buffer. Finally, the level of OsS1Fa1 was analyzed by Western blotting with anti-OsS1Fa1 antibody. To estimate the effect of MG132 on OsS1Fa1 in vivo, 2-week-old transgenic Arabidopsis seedlings or 7-d-old rice seedlings grown on MS medium were treated with 50 µM MG132 for 15 h. Leaf samples were ground in liquid nitrogen, and equal amounts of total protein were analyzed by Western blotting with anti-OsS1Fa1 antibody.

## 3. Results

### 3.1. Expression of OsS1Fa1 Is Upregulated by Drought Stress

**Figure 1:**
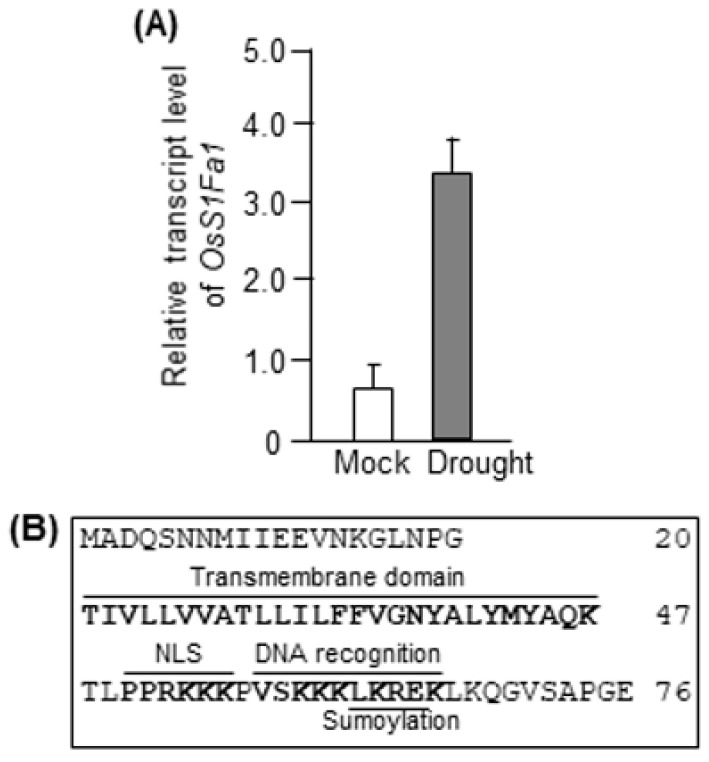
Analysis of *OsS1Fa1* transcript levels and the OsS1Fa1 amino acid sequence. (**A**) RT-qPCR analysis of *OsS1Fa1* expression in rice seedlings under drought stress. Wild-type (WT) seedlings were grown for 20 d under well-watered conditions by maintaining equal-sized seedlings in pots. Irrigation was withheld on day 20 for the next 19 d. Transcript levels of *OsS1Fa1* were examined by RT-qPCR using gene-specific primers. Data represent mean ± standard deviation (SD; *n* = 3). (**B**) The deduced amino acid sequence of OsS1Fa1. Conserved motifs are indicated in bold.

To identify the small protein involved in the drought stress response in rice, we chose *S1Fa* (LOC_Os04g33420), as it has not yet been functionally characterized in rice and is highly conserved among monocots and dicots [14]. To perform the drought stress treatment, wild-type (WT) seedlings were grown in a pot for 20 d under well-watered conditions. On day 20, irrigation was withheld for the next 19 d. To determine the role of OsS1Fa1 in drought stress response, we first investigated the expression pattern of the *OsS1Fa1* gene by quantitative real-time PCR (RT-qPCR). The expression of *OsS1Fa1* was significantly induced in rice leaves following the dehydration treatment (Figure 1A). Interestingly, the deduced amino acid sequence of OsS1Fa1 contained four possible functional domains: transmembrane domain, NLS, DNA recognition α-helix, and sumoylation motif (Figure 1B). Thus, despite its small size (76 aa), OsS1Fa1 could perform multiple functions in different subcellular organs.

### 3.2. OsS1F1a Transcript and the Encoded Protein Exhibit Organ-Specific Expression

**Figure 2:**
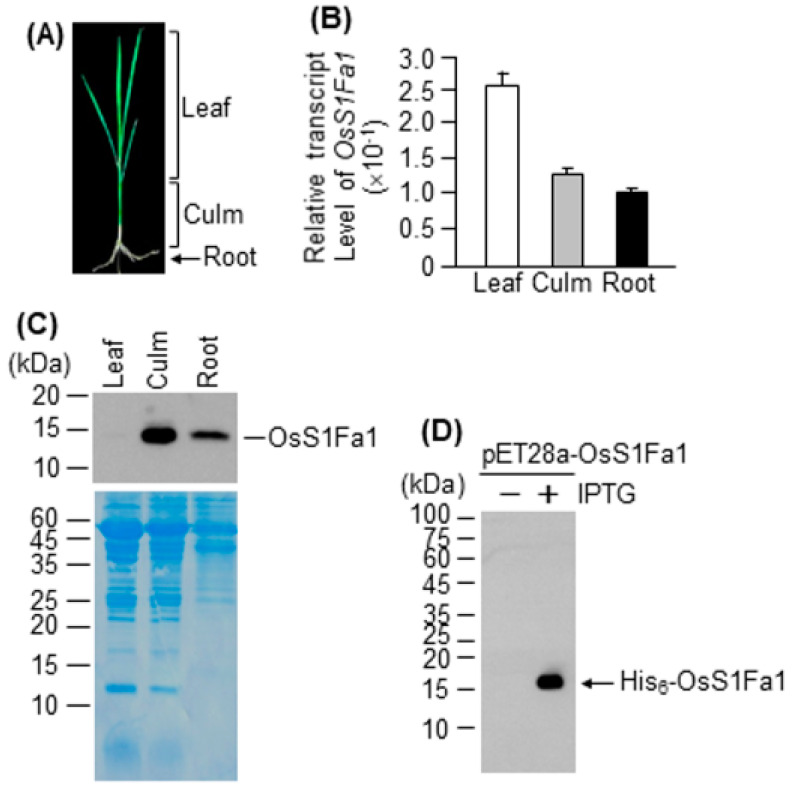
Analysis of *OsS1Fa1* expression in rice tissues and detection of the His-tagged recombinant proteins in *E. coli*. (**A**) Photograph of a 3-week-old rice seedling showing the leaf, culm, and root tissues used for total RNA isolation. (**B**) Expression analysis of *OsS1Fa1* in rice tissues by RT-qPCR. Data represent mean ± SD (*n* = 3). (**C**) Western blot analysis of OsS1Fa1 in rice tissues using anti-OsS1Fa1 antibody. Total proteins were extracted from the same samples as those used in (**B**). The corresponding protein gel is shown below. (**D**) Detection of the His_6_-OsS1Fa1 recombinant protein in *E. coli* extracts using anti-OsS1Fa1 antibody. The recombinant protein was detected in *E. coli* before and after the addition of IPTG.

Next, we evaluated the expression level of *OsS1Fa1* in the leaf, culm, and root tissues of 14-d-old rice seedlings (Figure 1A). The *OsS1Fa1* expression was higher in leaves than in culms and roots (Figure 2B). To investigate the OsS1F1a protein levels, we produced an anti-OsS1Fa1 antibody using the recombinant His_6_-OsS1Fa1 protein, which was produced in *E. coli* and purified by nickel affinity chromatography. The results of Western blotting showed that the OsS1Fa1 protein level was very low in leaves (unlike its transcript level) but was detectable in the culm and root (consistent with its transcript level) (Figure 2C). The molecular weight (MW) of OsS1Fa1, based on its amino acid sequence, was estimated at approximately 8.36 kDa; however, on the Western blot, the OsS1Fa1protein was detected at approximately 14.0 kDa (Figure 2C).

We also examined the affinity of anti-OsS1Fa1 antibody to bind to OsS1Fa1 in the crude extracts of *E. coli* expressing *His_6_-OsS1Fa1*. The results showed that the anti-OsS1Fa1 antibody specifically recognized OsS1Fa1 (Figure 2D). The size of the His_6_-OsS1Fa1 protein was approximately 15.5 kDa on the Western blot, which was greater than that predicted based on its amino acid sequence (9.02 kDa) (Figure 2D).

**Figure 3:**
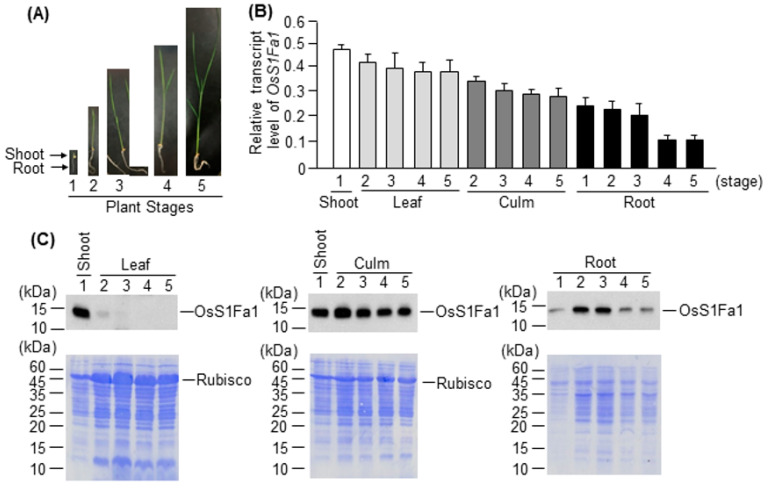
Analysis of *OsS1Fa1* transcript level and the corresponding protein level in different tissues of rice seedlings at different development stages. (**A**) Photographs showing rice seedlings at five different developmental stages. (**B**) RT-qPCR analysis of *OsS1Fa1* expression in the leaf, culm, and root tissues of rice seedlings at different developmental stages. Stage 1, one leaf (7-d-old seedlings); stage 2, two leaves (10-d-old seedlings); stage 3, three leaves (13-d-old seedlings); stage 4, four leaves (19-d-old seedlings); stage 5, five leaves (21-d-old seedlings). Data represent mean ± SD (*n* = 3). (**C**) Western blot analysis of the OsS1Fa1 protein using anti-OsS1Fa1 antibody. Total proteins were extracted from the same samples used in (**B**). Corresponding protein gels are shown below.

Because the OsS1Fa1 protein level in the leaf tissue was quite low, despite the high *OsS1Fa1* transcript level, we evaluated the expression level of *OsS1Fa1* in the shoot, leaf, culm, and root of rice seedlings at five different growth stages (1–5) (Figure 3A). The *OsS1Fa1* transcript level in the shoot at stage 1 was approximately 1.25-fold higher than that in leaf tissues at stages 2–5 (Figure 3B), and in the culm and root tissues, the *OsS1Fa1* transcript levels decreased slightly during growth. We also evaluated the OsS1Fa1 protein level in different tissues of rice seedlings at different growth stages. The level of OsS1Fa1 in leaves was lower at stage 2 than at stage 1, and the protein was undetectable in leaves at stages 3 to 5. However, in the culm and root tissues, the OsS1Fa1 protein level was somehow proportional to its gene transcript level at all growth stages (Figure 3C).

### 3.3. OsS1Fa1 Overexpression in Arabidopsis Enhances Drought Stress Tolerance

**Figure 4:**
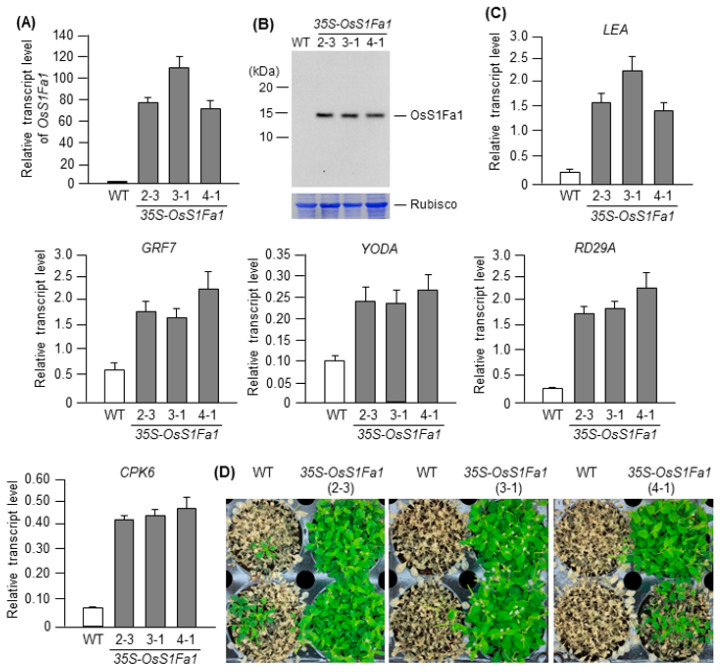
Generation and characterization of transgenic Arabidopsis lines overexpressing *OsS1Fa1*. (**A**) RT-qPCR analysis of *OsS1Fa1* expression in WT and transgenic Arabidopsis plants using gene-specific primers. Data represent mean ± SD (*n* = 3). (**B**) Western blot analysis of the OsS1Fa1 protein in WT and transgenic plants using anti-OsS1Fa1 antibody. Total proteins were isolated from the same samples as those used in (**B**). Rubisco was used as the loading control. (**C**) Expression analysis of *LEA*, *GRF7*, *YODA*, *RD29A*, and *CPK6* in WT and transgenic plants by RT-qPCR using gene-specific primers. Total RNA samples used in this experiment were the same as those used in (**A**). Data represent mean ± SD (*n* = 3). (**D**) Drought tolerance assay.

Because drought treatment increased the expression level of *OsS1Fa1* in rice (Figure 1A), we hypothesized that OsS1Fa1 acts as a positive regulator of drought stress tolerance. To test this hypothesis, we constructed and introduced the *35S-OsS1Fa1* plasmid in Arabidopsis and analyzed the *OsS1Fa1* transcript and OsS1Fa1 protein levels in transgenic Arabidopsis lines by RT-qPCR and Western blotting, respectively. The *OsS1Fa1* gene was expressed to high levels in three different transgenic lines, and the results of Western blotting were consistent with those of RT-qPCR, although a slight difference was observed between *OsS1Fa1* transcript and OsS1Fa1 protein levels (Figure 4A,B). Next, we determined the transcript levels of drought-responsive genes, including *Late Embryogenesis Abundant* (*LEA*), *Growth Regulating Factor 7* (*GRF7*), *YODA*, a mitogen-activated kinase kinase kinase (MAPKKK) gene, *Response-to-Dehydration 29A* (*RD29A*), and *Calcium-dependent protein kinase 6* (*CPK6*), in transgenic *OsS1Fa1* overexpression lines. Expression levels of all drought-responsive genes were higher in transgenic lines than in WT plants (Figure 4C). Next, we examined the drought stress tolerance of transgenic *OsS1Fa1* overexpression lines. Seeds of the WT and transgenic lines were cold-stratified on wet soil for 3 d, and the emerged seedlings were grown at 22 °C without watering for 28 d. After the drought stress treatment, plants were rewatered for 5 d and then photographed. The results showed that the *OsS1Fa1*-overexpressing plants showed higher rate of plant survival than the WT plants (Figure 4D). Thus, these data suggest that OsS1Fa1 enhances plant survival under drought.

### 3.4. OsS1Fa1 Degradation Is Influenced by Proteasome Inhibition

**Figure 5:**
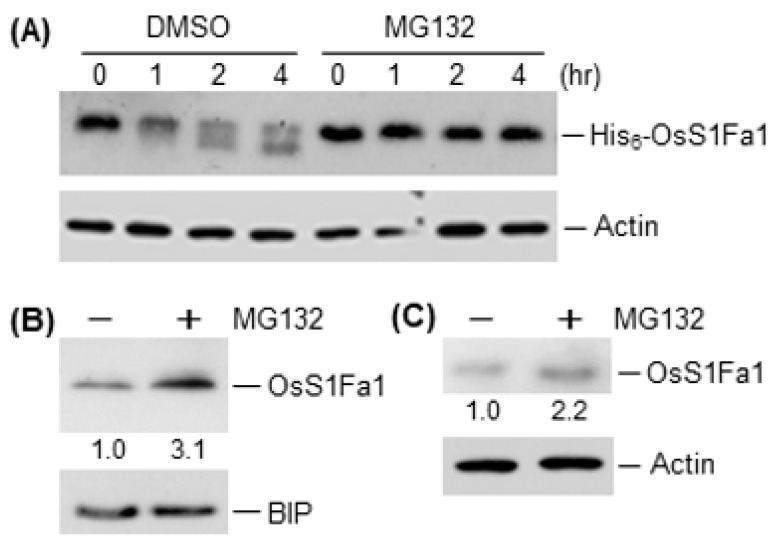
Effect of a proteasome inhibitor on the OsS1Fa1 protein level. (**A**) Cell-free degradation assay of OsS1Fa1. Rice leaf extracts were mixed with purified His_6_-OsS1Fa1 and treated with 50 µM MG132 (26S proteasome inhibitor) or 2% dimethyl sulfoxide (DMSO; solvent for the inhibitor; control) for the indicated time periods. OsS1Fa1 levels were determined by Western blotting using anti-OsS1Fa1 antibody, with known concentrations of actin serving as loading controls. (**B**,**C**) Proteasome pathway-mediated OsS1Fa1 degradation in vivo. Rice seedlings (**B**) and *OsS1Fa1*-overexpressing Arabidopsis plants (**C**) were treated with 50 µM MG132. After overnight incubation, the OsS1Fa1 protein concentration was assessed by Western blotting using anti-OsS1Fa1 antibody, with known concentrations of actin or binding protein (BIP) serving as loading controls. Values below each lane indicate relative intensities. All experiments were repeated three times, with similar results.

The high transcript and low protein levels of OsS1Fa1 during leaf development (Figure 2B,C and Figure 3B,C) suggest that the stability of OsS1Fa1 is regulated at the post-translational level. To test this possibility, we examined the cell-free degradation of OsS1Fa1 using the total protein extract from rice leaves and purified His_6_-OsS1Fa1 in the presence or absence of a 26S proteasome inhibitor, MG132. OsS1Fa1 degradation was substantially delayed with the proteasome inhibitor (Figure 5A). The effect of MG132 on OsS1Fa1 levels in rice seedlings and *OsS1Fa1*-overexpressing Arabidopsis plants was also assessed. The OsS1Fa1 levels increased in both rice seedlings and transgenic Arabidopsis plants after direct treatment with MG132, as shown by Western blotting with anti-OsS1Fa1 antibody (Figure 5B,C). These results indicate that OsS1Fa1 can be degraded by the 26S proteasome complex.

## 4. Discussion

In this study, we demonstrate that the rice small protein OsS1Fa1 enhances drought tolerance in Arabidopsis. Recent studies have improved our understanding of the molecular genetic mechanisms underlying drought resistance in plants, thus facilitating the development of drought tolerant crops. However, most of these studies investigated large transcripts and proteins. Recently, several research groups reported the role of small proteins and peptides in plant development, nutrient assimilation, and environmental stress response [22,23,24,25,26]. This has led to a large-scale investigation of small proteins and peptides via bioinformatic analysis of genomic, transcriptomic, and proteomic data [27,28].

Rice is a semi-aquatic plant species that requires standing water for proper growth and development. Thus, drought is one of the major abiotic stresses that directly influences plant growth and productivity in rice. Here, we explored the role of OsS1Fa1 in the drought stress response. A previous study showed that spinach S1Fa is expressed to higher levels in etiolated seedlings and roots than in green leaves [13]. In the current study, OsS1Fa1 was highly expressed in green leaves and culms; however, its expression was relatively low in roots (Figure 2B, Figure 3B). These data suggest that the expression pattern of *S1Fa* genes in monocots, including rice, is different from that in dicots such as spinach.

Protein mobility shift during SDS-PAGE fractionation can occur for several reasons. First, it is caused by chemical modifications of proteins such as phosphorylation [29], glycosylation [30], hydroxylation [31], methylation [32], and ubiquitination [33,34]. The acidity and hydrophobicity of proteins are other factors that could influence protein mobility [35,36]. In our data, the MW of native OsS1Fa1 and recombinant His_6_-OsS1Fa1 proteins in SDS-PAGE is approximately 5.64 and 6.30 kDa larger than its predicted MW, respectively (Figure 2C,D). OsS1Fa1 contains a transmembrane domain (Figure 1B), thus suggesting that its migration might be affected by a hydrophobic surface. Further examination of the transmembrane domain of mutant OsS1Fa1 proteins is required to find out the cause of the mobility shift. It is also possible that OsS1Fa1 is modified by chemicals. Mass spectrometry analysis by using gel electrophoresis liquid chromatography-mass spectrometry can also be applied to find a clue.

A number of small proteins and peptides, such as CLAVATA3/EMBRYO-SURROUNDING REGION-RELATED 25 (CLE25), INFLORESCENCE DEFICIENT IN ABSCISSION (IDA), and IDA-LIKE 1, are involved in the response to abiotic stresses such as drought and salt [18,37,38,39,40]. Our results showed that *OsS1Fa1* expression was induced under drought stress (Figure 1A). Consistent with this result, overexpression of *OsS1Fa1* in Arabidopsis increased the survival rate of transgenic plants under drought (Figure 4D). Additionally, *OsS1Fa1* overexpression in Arabidopsis upregulated the expression of drought-responsive genes (Figure 4E). These data indicate that OsS1Fa1 protects plants against drought stress. Despite the high transcript levels of *OsS1Fa1* in leaves during development, the level of the OsS1Fa1 protein in leaf tissues was very low (Figure 2B,C, Figure 3B,C), suggesting that the stability of OsS1Fa1 is regulated at the post-translational level. We, therefore, determined the concentration of OsS1Fa1 in transgenic Arabidopsis plants carrying the *35S-OsS1Fa1* construct as well as in rice seedlings. Treatment with the proteasome inhibitor MG132 increased the concentration of OsS1Fa1 and delayed its degradation (Figure 5A–C). In addition, the computer program QUARK predicted several ubiquitination sites in OsS1Fa1 (Table S3). Taken together, these results indicate that the stability of OsS1Fa1 in vivo is regulated by the proteasome complex after polyubiquitination.

The binding of spinach S1Fa to the *rpsl* gene promoter in the nucleus [11] suggests that S1Fa functions as a transcription factor. In this study, we found a putative sumoylation motif in the DNA recognition α-helix domain of OsS1Fa1 (Figure 1B). Increasing evidence shows that sumoylation changes the conformation of the target protein, thereby affecting its subcellular localization, stability, and activity as well as interactions with other proteins [41,42,43,44]. This suggests that the transcriptional activation function and stability of OsS1Fa1 can be controlled by sumoylation. In addition, the presence of a transmembrane domain in OsS1Fa1 strongly suggests that OsS1Fa1 functions in the membrane and potentially participates in cellular processes as an interaction partner of other membrane proteins. Further subcellular localization analysis of the OsS1Fa1 protein and the identification of nuclear and cytoplasmic membrane-localized OsS1Fa1-interacting proteins will help to further understand the function of OsS1Fa1 in the cell.

MicroProteins are small proteins that interact with and modulate the activity of large proteins [45]. Recently, an improved algorithm capable of processing poorly annotated genomes and advanced translatomics was employed to predict small proteins and their interacting partners [46,47]. This strategy can be used to identify the function of the small protein itself or the effects of small proteins on their interacting partners. MicroProteins also act as a regulatory tool. For example, in Arabidopsis, two B-box-containing microProteins, microProtein1a (miP1a) and miP1b, regulate CONSTANS (CO) to control flowering time [48]. In addition, the overexpression of the synthetic Hd1 B-box domain in rice interferes with the endogenous function of the CO homolog, Hd1 [49]. Small proteins are also known to regulate fundamental plant processes such as growth and stress response [47] and therefore can be used for crop bioengineering. Together, these studies suggest that OsS1Fa1 interacts with target proteins via its NLS and transmembrane domain and can be used to develop drought tolerance crops.

In summary, *OsS1Fa1* expression is induced by drought, and overexpression of *OsS1Fa1* in Arabidopsis not only enhances drought stress tolerance but also increases the expression of drought tolerance-related genes. In addition, the stability of the OsS1Fa1 protein is post-translationally modulated. Further studies are needed to investigate the potential roles of the conserved motifs of OsS1Fa1 in its subcellular localization. Elucidation of the ubiquitination and sumoylation of OsS1Fa1 in vivo and the effects of *OsS1Fa1* overexpression on drought stress tolerance are also required for understanding the role of OsS1Fa1. In addition to these, identification of OsS1Fa1-interacting proteins would bring another insight into the understanding of the function of OsS1Fa1 in drought stress response. Together, these studies will elucidate the role of OsS1Fa1 in plant development and stress responses.

## Supplementary Materials

The following are available online at https://www.mdpi.com/article/10.3390/plants10102181/s1, Table S1: Putative ubiquitination sites of OsS1Fa1 identified using the computational Prediction of protein Ubiquitination sites with a Bayesian Discrimination Method (BDM-PUB), Table S2: Primers used for plasmid construction, Table S3: Primers used to perform quantitative real-time PCR (RT-qPCR).
