# Supplementary material for: Overexpression of Rice OsS1Fa1 Gene Confers Drought Tolerance in Arabidopsis
Source: Plants (Basel). 2021 Oct 14;10(10):2181. doi: 10.3390/plants10102181 (PMC8541125; doi:10.3390/plants10102181)
Supplement: Supplementary file 1 [file plants-10-02181-s001.zip › plants-1388028-supplementary.pdf]

Supplementary Materials

Table S1. Primers used for plasmid construction.

Primer name	Primer sequence (5'→3')
OsS1Fa1_F	CAGTGAATTCATGGCGGATCAGTCGAACAACA
OsS1Fa1_R	CAGTCTCGAGCTCTCCGGGTGCAGAAACC

Table S2. Primers used to perform quantitative real-time PCR (RT-qPCR).

Name	Primer sequence (5'→3')
UBQ5_F	ACCACTTCGACCGCCACTACT
UBQ5_R	ACGCCTAAGCCTGCTGGTT
OsS1Fa1_F	CATGATCATCGAGGAGGTCA
OsS1Fa1_R	TCAGCTTTTCCCTCTTCAGC
LEA_F	GCAATCAAGAACAAGGCACA
LEA_R	CAGTGCGAAGCCCTAAAGT
GRF7_F	CGCATCTATTCCTGTTCCTT
GRF7_R	ATCTTCCCGGCTCTAAATCT
YODA_F	AGACCCGGTTTTTCAACTCC
YODA_R	TTTGACTCGAACGGGTTAGG

Table S3. Putative ubiquitination sites of OsS1Fa1 identified using the computational Prediction of protein Ubiquitination sites with a Bayesian Discrimination Method (BDM-PUB).

Peptide†	Position	Score
ALYMYAQKTLPPRKK	47	0.64
QKTLPPRKKKPVSKK	53	2.35
KTLPPRKKKPVSKKK	54	2.14
TLPPRKKKPVSKKKL	55	2.91
RKKKPVSKKKLKREK	59	4.06
KKKPVSKKKLKREKL	60	3.17
KKPVSKKKLKREKLK	61	2.37
PVSKKKLKREKLKQG	63	3.64
KKKLKREKLKQGVSA	66	1.71
KLKREKLKQGVSAPG	68	1.65

†Lysine (K) residues, which covalently bind to ubiquitin, are indicated in red font.
